# Evidence of within-facility patient–patient *Clostridiodes difficile* infection spread across diverse settings

**DOI:** 10.1017/S0950268822001893

**Published:** 2022-12-11

**Authors:** Samuel Justice, Daniel K. Sewell, Haomin Li, Aaron C. Miller, Philip M. Polgreen

**Affiliations:** 1 Department of Statistics, University of Iowa, Iowa City, USA; 2 Department of Biostatistics, University of Iowa, Iowa City, USA; 3 Department of Internal Medicine, University of Iowa, Iowa City, USA; 4 Department of Epidemiology, University of Iowa, Iowa City, USA

**Keywords:** *Clostridioides difficile*, hospital-acquired (nosocomial) infections, infectious disease epidemiology

## Abstract

Previous studies have suggested that a hospital patient's risk of developing healthcare facility-onset (HCFO) *Clostridioides difficile* infections (CDIs) increases with the number of concurrent spatially proximate patients with CDI, termed CDI pressure. However, these studies were performed either in a single institution or in a single state with a very coarse measure of concurrence. We conducted a retrospective case-control study involving over 17.5 million inpatient visits across 700 hospitals in eight US states. We built a weighted, directed network connecting overlapping inpatient visits to measure facility-level CDI pressure. We then matched HCFO-CDIs with non-CDI controls on facility, comorbidities and demographics and performed a conditional logistic regression to determine the odds of developing HCFO-CDI given the number of coincident patient visits with CDI. On average, cases' visits coincided with 9.2 CDI cases, which for an individual with an average length of stay corresponded to an estimated 17.7% (95% CI 12.9–22.7%) increase in the odds of acquiring HCFO-CDI compared to an inpatient visit without concurrent CDI cases or fully isolated from both direct and indirect risks from concurrent CDI cases. These results suggest that, either directly or indirectly, hospital patients with CDI lead to CDIs in non-infected patients with temporally overlapping visits.

## Introduction

*Clostridioides difficile* is a significant cause of morbidity and mortality [1, 2]. While community-onset *C. difficile* infections (CDIs) are increasing, healthcare facility-onset CDIs (HCFO-CDIs) still comprise the majority of cases. Also, HCFO-CDI is associated with three times the cost per patient compared to similar non-HCFO-CDI patients [3]. Individual risk factors include increased age [4], use of proton-pump inhibitors [5], acid suppressing drugs and antibiotics [6].

Several studies have found *C. difficile* contamination on healthcare worker hands and surfaces in healthcare facilities [7–12]. Also, significant spatiotemporal clustering of CDI cases [13] provides evidence of direct or indirect transmission of *C. difficile* between patients. In addition, the number of infected or colonised individuals in close spatial and temporal proximity to a given patient, known as ‘pressure’, has been found to be a significant risk factor for infection from several other pathogens such as methicillin-resistant *Staphylococcus aureus* [14], vancomycin-resistant Enterococcus [15], multi-antimicrobial-resistant *Acinetobacter* spp, carbapenem-resistant *Pseudomonas aeruginosa* [16] and multi-antimicrobial-resistant *Acinetobacter baumannii* [17]. CDI pressure is an important risk factor at the very local level of room sharing or adjacent rooms [18], as well as at the unit level [19, 20], and whole genome sequencing (WGS) has provided evidence of links between cases connected by a common unit [21].

*C. difficile* spores persist in the environment, and lingering spores can lead to secondary infections [22]. In addition, a WGS study showed that 22% of *C. difficile* transmission events occurred between units, and the authors identified potential super-spreaders affecting CDIs across the hospital [23]. This suggests that in addition to local CDI pressure, the movement of patients throughout a healthcare facility and peripatetic healthcare workers visiting multiple units may lead to broader CDI pressure on the facility level. Indeed, Miller *et al*. demonstrated that hospital-wide CDI pressure is a risk factor for HCFO-CDI [24]. While previous studies of CDI pressure were limited to a small number of healthcare facilities over a short time period, Miller *et al*.'s study on hospital-level CDI pressure considered all hospitals in California. However, the measure of CDI pressure was limited to temporally aggregating CDI cases to 3-month periods. The current study expands on this work in two important ways. First, we devised a method for determining CDI pressure that is specific to each patient's visit rather than relying on coarse temporal aggregation. Second, we measured CDI pressure over a much larger geographic area and over a longer time period in order to determine if patient-level risk due to hospital-level CDI pressure can be generalised across different settings in different states.

## Methods

### Data

This study utilised data from the State Inpatient Databases (SID), a family of administrative claims database available through the Healthcare Cost and Utilization Project (HCUP). The SID contain information regarding discharge abstracts for 100% of inpatient visits to non-federal hospitals for specific states and years. The study included data from the SID for eight different states: Arkansas from 2005 to 2014, Arizona from 2005 to 2007, Iowa from 2009 to 2015, Nebraska from 2009 to 2014, North Carolina from 2005 to 2010, Utah from 2005 to 2012, Vermont from 2011 to 2015 and Wisconsin from 2013 to 2015. There was a total of 16 876 332 inpatient visits between these eight states. CDI cases were identified as visits with either an *International Classification of Disease, Ninth Revision, Clinical Modification* (ICD-9-CM) diagnosis code of 008.45 or an *International Classification of Disease, Tenth Revision, Clinical Modification* (ICD-10-CM) diagnosis code of A04.7. Our main interest concerned HCFO-CDI, i.e. patients who were infected with CDI during their stay at a hospital.

### Analysis

#### Cohort

We built a decision tree to classify patient visits with a CDI as either an HCFO-CDI or community acquired/indeterminate. The full decision tree providing details on this classification is given in Appendix A, and is built on the definitions provided in [25–27]. To ensure comparability between cases and controls, inclusion criteria applied to the HCFO-CDIs (such as length of stay greater than 3 days) was also applied to the controls. Each of the HCFO-CDIs was matched with a non-CDI control based on age, gender, hospital, diagnoses and admission year. For the purposes of matching, patients were binned into 10-year age groups (0–10, 11–20, 21–30, etc.). Patients were matched sequentially based on their diagnoses, at each step using a coarser diagnosis categorisation. They were first matched on Diagnosis-Related Group (DRG), a patient classification system consisting of 467 categories; if there was no match here, they were next matched on their primary Clinical Classifications Software (CCS) code, of which there are 285 distinct categories; finally, if both DRG and CCS code failed to provide a match, we used Major Diagnostic Category (MDC), which consists of only 25 diagnosis groups. If a particular HCFO-CDI could not be matched based on any of these three diagnosis systems (in addition to age, gender and hospital), this case was excluded from the cohort. When there were multiple matches for an HCFO-CDI based on the four criteria, a single match was randomly selected from among them. Once a control was matched to an HCFO-CDI, it could not be matched to any other HCFO-CDIs. Of the 99.8% HCFO-CDIs which were successfully matched to a control, approximately 94% were matched based on DRG, 5% were matched on CCS code and 1% were matched on MDC.

#### CDI potential transmission network

We constructed a weighted, directed network for each state, where every vertex in the network represented either a CDI case (including cases not classified as HCFO-CDIs) or a matched control in our cohort. These networks captured the probability that a patient with CDI had the opportunity to cause a HCFO-CDI in another patient. Generally, this consisted of looking at all pairs of hospital visits, determining if these pairs coincided in time and occurred at the same facility, and considering if one patient had CDI and the other entered the hospital without CDI. More specifically, for each state's network there was a directed edge present from case *i* to case/control *j* if *i*'s visit intersected with *j*'s visit. Thus, edges could only emanate from CDI cases, but they could be received by both cases and controls. The HCUP SID provides a linking variable to connect hospital visits with patients, alongside the hospital of each visit, the admission and discharge month of each visit, the length of stay for each visit and the number of days between visits. To protect patient identities, the precise admission and discharge days are not provided, and therefore it is in general impossible to be completely certain that two patients had intersecting visits. To remedy this issue, we devised a method for computing the probability that the *i*th patient's visit intersected with the *j*th patient's visit based on their respective facilities, admission/discharge months and lengths of stay, and we then took this probability to be the weight for the edge from *i* to *j*. Upon constructing each state's network, the in-degree for patient *i* was computed by the sum of the weighted edges received by *i*. Due to the methods used to construct the weighted network, the in-degree for patient *i* equals the expected number of other patients at the same facility with a CDI (HCFO or otherwise) whose visits overlapped with that of *i*. See Appendix B for a detailed description of how the network was constructed for each state.

#### Statistical analysis

We employed conditional logistic regression on our matched cohort using a 0–1 indicator for a HCFO-CDI as the response variable. We used each patient's in-degree as the primary covariate of interest to capture CDI pressure. We added patient length of stay as a term in the model to account for the fact that HCFO-CDI patients tended to have prolonged hospital stays (which in turn inflated their in-degrees). We used empirical logit plots to determine if a transformation was required to meet modelling assumptions, and used AIC to determine whether or not an interaction term between in-degree and length of stay was necessary. The interpretation of the (exponentiated) coefficient for in-degree can be interpreted in two ways. First, it can refer to the change in the odds of a HCFO-CDI due to each additional concurrent CDI case; this is the way we focus on in the Results section. Alternatively, it can easily be shown mathematically to be equivalent to the change in the odds of a HCFO-CDI due to each concurrent CDI case above the average number of concurrent CDI cases at the facility an individual's visit corresponds to. In this way, we can understand a positive statistically significant regression coefficient for in-degree to have a global interpretation in the sense that such an effect is not determined by the size and overall prevalence of any specific facility. Rather, in-patients staying at a facility experiencing a higher (lower) than average number of CDI cases is at an increased (decreased) risk of HCFO-CDI, and that this risk is amplified (dampened) by the degree to which a facility is above (below) its average prevalence.

To control for differences in patient characteristics not captured by our matching scheme, we also included 29 indicator variables for various Elixhauser comorbidities (see Table 2). We further incorporated temporal sine and cosine terms into the model to account for seasonality with regards to CDI. All statistical analyses were conducted using the R programming language [28], using the R packages dbplyr [29], survival [30] and Matrix [31].

#### Sensitivity analysis

If the estimated effect of CDI pressure were solely due to inflated in-degrees caused by longer lengths of stay, making controls and cases have similar in-degree distributions ought to nullify the effect of CDI pressure in our analysis. To investigate the robustness of our results to issues related to length of stay, we performed a sensitivity analysis. Instead of including length of stay as a covariate, we adjusted the networks to account for differing length of stay distributions between cases and controls. Specifically, we calculated the difference in mean length of stay between the HCFO-CDIs and controls in our cohort; then when computing the edges in each network for the HCFO-CDIs, we decreased their lengths of stay by this difference. If any of the resulting lengths of stay were less than 1 day, we took them to be 1 day (adjusting the corresponding discharge months if necessary). We then ran a conditional logistic regression model using in-degree as calculated from the resulting modified networks; as before, Elixhauser comorbidities and seasonality terms were also included as covariates.

## Results

**Fig. 1.**
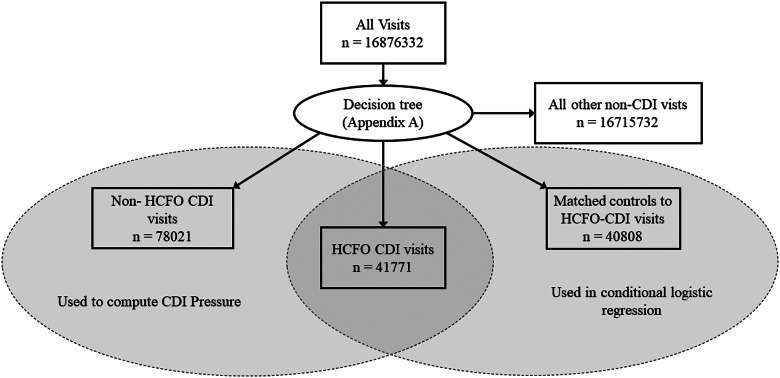
Number of inpatient hospital visits that were classified as HCFO-CDIs, all other CDIs, matched controls and all other non-CDI cases.

Using the classification tree shown in Appendix A, we identified a total of 40 808 HCFO-CDIs between the eight states over the span of each of their data. This total excluded 963 HCFO-CDIs for which we were not able to find a matching control, although such patients still contributed to CDI pressure in our constructed networks, as did all other CDIs. Table 1 provides a breakdown of the total numbers of hospitals, admissions and HCFO-CDIs by state. Figure 1 provides a consort schematic showing the total numbers used in our analyses.

**Table 1.** Total numbers of hospitals, admissions and HCFO-CDIs for each of the eight states included in the study

State	Years	Number of hospitals	Total admissions	Total HCFO-CDIs
Arkansas	2005–2014	110	3 891 638	6209
Arizona	2005–2007	80	2 244 437	9273
Iowa	2009–2015	117	1 805 473	3109
Nebraska	2009–2014	89	1 258 818	1674
North Carolina	2005–2010	108	4 361 475	12 071
Utah	2005–2012	51	1 862 873	4917
Vermont	2011–2015	14	214 553	397
Wisconsin	2013–2015	148	1 820 219	3158

**Fig. 2.**
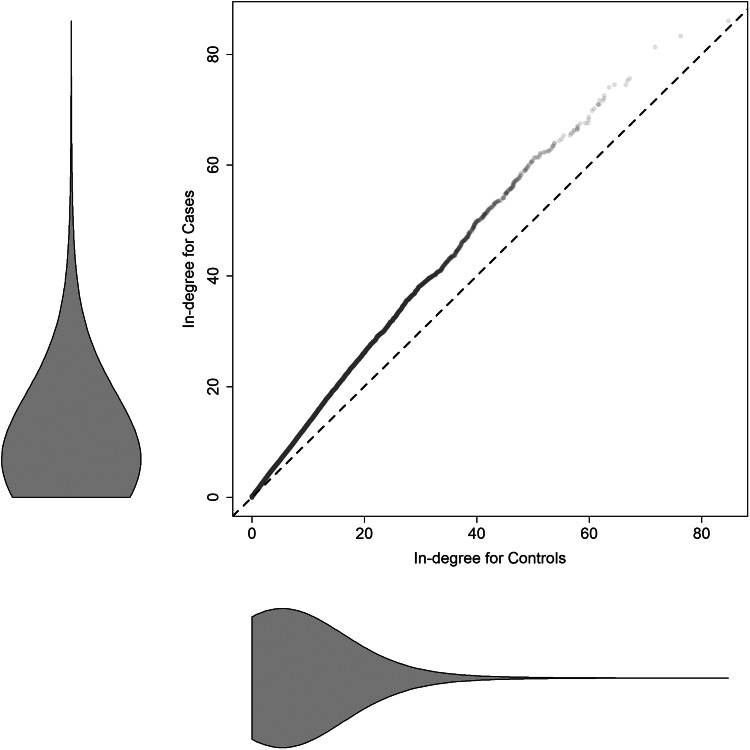
QQ-plot comparing the distributions of the in-degree, i.e. the expected number of concurrent CDI cases, for HCFO-CDI cases (vertical) and controls (horizontal). The dashed line serves as a reference showing where we would expect the points to lie should the distributions be equal. Violin plots show the marginal in-degree distributions.

The mean (s.d.) numbers of coincident CDI cases were 6.9 (7.1), 9.2 (9.0) and 8.4 (8.4) for the controls, HCFO-CDI cases and HCFO-CDI cases after adjusting for length of stay differences respectively. Figure 2 compares the distributions of the expected number of coincident CDI cases for HCFO-CDI cases and controls using a qq-plot. The dashed line corresponds to equivalent distributions, and from this we can see that every quantile for the cases is larger than that of the controls (equivalent plots adjusting cases for length of stay showed similar results). Marginal distributions for both cases and controls are provided as violin plots in the margins.

**Fig. 3.**
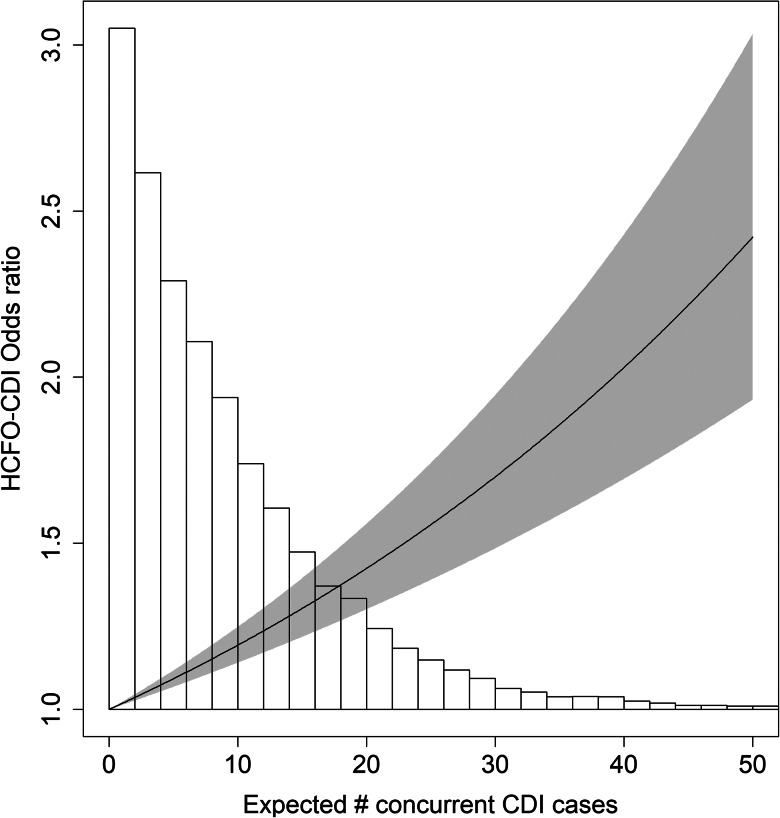
Odds ratio (vertical axis) corresponding to an increase in the expected number of concurrent CDI cases (horizontal axis). The pointwise confidence band is shaded grey. The background histogram shows the distribution of the in-degree for cases.

Table 2 displays the results of the conditional logistic regression model for our primary analysis. While it was clear that in-degree had a strong linear relationship with the response (on the logit scale), length of stay required a log transformation to satisfy model assumptions. Using AIC, we determined that the interaction term between in-degree and (log) length of stay was an important predictor of HCFO-CDI status. The coefficients for length of stay and in-degree were both positive as expected, while the interaction term had a negative coefficient, implying that as the length of stay increases the risk associated with each additional concurrent CDI patient is attenuated. For a patient with the average length of stay of 4.53 days, each additional concurrent CDI case (i.e. in-degree) was associated with a 1.8% increase in the odds of acquiring CDI (*P* value < 0.001; 95% CI 1.3–2.2%). On average, the in-degree of cases was 9.2 leading to a 17.7% (95% CI 12.9–22.7%) increase in the odds of a HCFO-CDI compared to an inpatient visit without concurrent CDI cases or the case where the patient is fully isolated from both direct and indirect risks from concurrent CDI cases. Figure 3 visualises the change in the odds ratio due to in-degree for an average length of stay, holding all other variables constant. Our sensitivity analysis regarding length of stay provided stronger results, yielding cases having a mean in-degree of 8.4, leading to a 22% (*P* value < 0.001; 95% CI 20–25%) increase in the odds of HCFO-CDI compared to an inpatient visit without concurrent CDI cases or the case where the patient is fully isolated from both direct and indirect risks from concurrent CDI cases.

**Table 2.** Results from the conditional logistic regression model evaluating the effect of CDI pressure on HCFO-CDI risk

Variable	Odds ratio/exponentiated regression coefficient (95% CI)	*P* value
Expected # of concurrent CDI cases	1.046 (1.036–1.056)	<0.001
log (Length of stay)	4.555 (4.366–4.752)	<0.001
(Expected # of concurrent CDI cases) × (log (length of stay))	0.982 (0.979–0.985)	<0.001
Sine	1.054 (1.030–1.079)	<0.001
Cosine	0.988 (0.965–1.011)	0.307
Alcohol abuse	0.887 (0.809–0.972)	0.010
Deficiency anaemias	0.996 (0.952–1.042)	0.874
Rheumatoid arthritis/collagen vascular diseases	1.052 (0.954–1.160)	0.307
Chronic blood loss anaemia	1.078 (0.958–1.212)	0.212
Congestive heart failure	0.943 (0.900–0.988)	0.134
Chronic pulmonary disease	0.903 (0.867–0.941)	<0.001
Coagulopathy	1.014 (0.949–1.083)	0.675
Depression	0.992 (0.932–1.055)	0.788
Diabetes, uncomplicated	0.849 (0.810–0.889)	<0.001
Diabetes with chronic complications	0.887 (0.823–0.956)	0.002
Drug abuse	0.808 (0.712–0.917)	<0.001
Hypertension	0.829 (0.799–0.859)	<0.001
Hypothyroidism	0.940 (0.887–0.996)	0.036
Liver disease	1.090 (0.991–1.199)	0.075
Lymphoma	1.180 (1.039–1.340)	0.011
Fluid and electrolyte disorders	1.428 (1.381–1.478)	<0.001
Metastatic cancer	0.776 (0.714–0.844)	<0.001
Other neurological disorders	0.989 (0.933–1.047)	0.701
Obesity	0.829 (0.773–0.889)	<0.001
Paralysis	0.902 (0.826–0.984)	0.021
Peripheral vascular disorders	0.956 (0.888–1.030)	0.240
Psychoses	0.982 (0.894–1.079)	0.707
Pulmonary circulation disorders	0.794 (0.719–0.877)	<0.001
Renal failure	1.163 (1.108–1.221)	<0.001
Solid tumour without metastasis	0.994 (0.905–1.091)	0.896
Peptic ulcer disease excluding bleeding	1.182 (0.592–2.359)	0.636
Valvular disease	0.875 (0.807–0.949)	0.001
Weight loss	1.154 (1.095–1.217)	<0.001
Admission year (reference: 2005)		
2006	0.916 (0.865–0.969)	0.002
2007	0.889 (0.840–0.942)	<0.001
2008	0.841 (0.779–0.909)	<0.001
2009	0.847 (0.787–0.911)	<0.001
2010	0.915 (0.851–0.984)	0.016
2011	1.016 (0.924–1.118)	0.741
2012	0.915 (0.831–1.006)	0.067
2013	0.749 (0.675–0.831)	<0.001
2014	0.832 (0.751–0.922)	<0.001
2015	0.818 (0.724–0.925)	0.001

## Discussion

The degree to which CDI may transmit in hospital settings has been a question of numerous investigations. Indeed, some whole-genome sequencing studies have questioned the transmissibility of CDI in healthcare settings, as genetic links between CDI cases have often been difficult to establish. However, our group and others have found evidence for the role of the hospital environment in the transmission of CDI [13, 18–20, 24]. In this study, we used coincident cases of CDI as a measure of CDI pressure and found that controlling for seasonality, temporal trends and patient comorbidities, for an individual with the average length of stay, each additional concurrent CDI patient was associated with a 1.8% increase in the odds of HCFO-CDI. The number of concurrent CDI patients can be large (for cases, this ranged from 0 to 86, with a mean of 9.2), implying that this represents a clinically significant increase in risk due to concurrent CDI patients at the same facility.

This study expands upon a body of research linking environmental infection pressure within healthcare settings to risk of transmission. Most prior investigations' results of CDI pressure have been based on a single or small group of connected healthcare facilities. While such investigations provide highly granular information for identifying and linking CDI cases, they are unable to characterise the extent to which the effect of CDI pressure may generalise to a broader range of healthcare settings and populations. In contrast, our study covered eight states and 717 hospitals across the USA, suggesting that the increase in risk of HCFO-CDI due to an increase in interhospital CDI pressure generalises across diverse settings. Miller *et al*. studied CDI pressure using state-wide discharge data from California to characterise a measure of CDI pressure across a wide range of healthcare settings [24]. However, this study, which relied on deidentified patient data, lacked the temporal granularity of exact admission dates required to establish epidemiological linkages between cases and CDI pressure. CDI pressure was defined at a hospital-quarterly level and thus may not directly correspond to the exact pressure that individual patients encounter. This study represents the first attempt to infer greater temporal granularity in CDI pressure using deidentified population-level discharge data. This approach may be utilised to study the effect of environmental infection pressure on other healthcare-associated infections and to better capture exposure risk on future investigations utilising such data sources. In addition, we carefully matched cases and controls, thereby accounting for demographics, the reason for the inpatient visit and, by matching on facility, any unmeasured facility-level confounder variables.

There were several limitations of this study. First, since the HCUP SID include only partial information on admission and discharge dates, we could only compute the expected, rather than actual, number of coincident CDI cases. Second, there was no information in the HCUP SID on asymptomatic infections, yet some evidence exists that suggests this may be an important transmission pathway [32]. Third, because spores may persist in the environment for up to 5 months, risk from past patients, unaccounted for in our analysis, may also contribute to a patient's risk of HCFO-CDI. Fourth, we do not have access lab results nor testing dates to confirm cases of CDI or the timing of symptoms relative to hospital admission. Thus, we cannot directly confirm CDI surveillance definitions or the exact length of stay prior to diagnosis. While some researchers have found strong sensitivity and specificity for identifying CDI cases [33, 34], others have found the sensitivity of ICD codes to be 50% or less, and the site-specific testing methods which was unavailable to us may have strong influence on this as well [35, 36]. Fifth, our data do not contain patient locations, and hence we are unable to account for the fact that some patients may be roommates while others may be situated in different wards or floors, nor do we know at the facility level the percentage of patients sharing their room with other patients. Additionally, because the dates of the patients' visits are inexact, we were unable to account for the number of days of overlap between two patients' visits.

Despite these limitations, our results provide further evidence for the role of hospital environments and the transmission of CDI. In our analysis, patients who were admitted to hospitals with a high probability of having an overlapping stay with other CDI patients were at increased risk for CDI. Moreover, as the expected number of other overlapping CDI cases increased so did their risk for CDI. As our study included a large number of institutions and geographic locations in the USA, our findings suggest that prior studies of CDI pressure within a small number of facilities were not anomalous, and that this effect, which still may prove to be heterogeneous across facilities and states, is nevertheless prevalent across a wide range of settings. Thus, our results continue to provide evidence for the role of institutional CDI pressure and risk for CDI.

## Figures and Tables

**Fig. A1.**
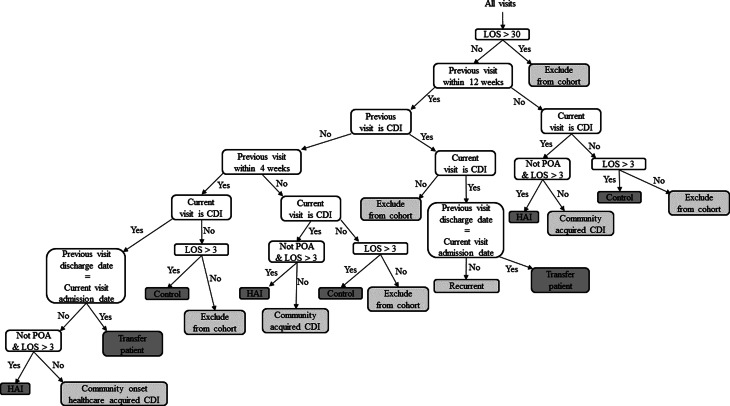
A tree illustrating how inpatient visits were classified in the study. LOS, length of stay; POA, present on admission tag or CDI was primary diagnosis; HCFO-CDI, healthcare-associated infection.

**Fig. A2.**
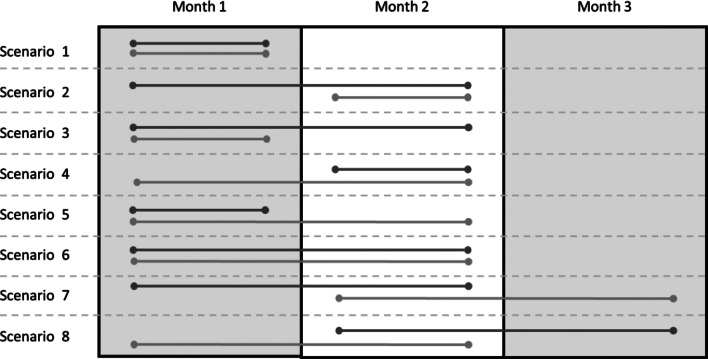
A depiction of the eight possible scenarios for how two inpatient visits could intersect in time at a given hospital.

## Data Availability

The data that support the findings of this study are available from the Healthcare Cost and Utilization Project (HCUP) State Inpatient Databases (SID) (https://www.hcup-us.ahrq.gov/sidoverview.jsp). HCUP data are available for a fee to all researchers following a standard application process and signing of a data use agreement.

## Appendix

Appendix A: Decision Tree for classifying HCFO-CDIs

Figure A1 displays the decision tree used to classify patient visits. Since only admission month, discharge month, length of stay and time between visits are given, visits with a length of stay (LOS) greater than 30 days made it too uncertain whether the time at which the patient was infected with *C. difficile* overlapped with other patient visits, and hence we excluded such patients. Following [25–27], any CDI visits with a LOS of 3 days or fewer were considered community acquired and were not included as cases (HCFO-CDIs) in our analysis; we therefore also excluded non-CDI visits with LOS ≤3. Any CDI visits which had either a primary CDI diagnosis or a present on admission tag associated with the CDI diagnosis were also considered to be community acquired, unless the patient had another hospital visit within the past 4 weeks, in which case they were considered to have a community-onset healthcare-associated CDI [25]; both types of patient visits were excluded from our cohort. We defined a readmission to be a CDI visit which had a previous CDI visit within the last 12 weeks. We did not include readmissions as cases in our statistical analysis, and hence we also excluded from our set of potential matched controls those non-CDI visits which had a previous CDI visit within the last 12 weeks. However, we treated CDI visits which were patient transfers specially, where a patient transfer was defined to be a visit with an admission date equal to a discharge date of a previous visit; see Appendix B for more details on transfer patients.

Appendix B: Constructing the CDI potential transmission networks

By looking at the location and time of each patient's visit, we constructed a network that corresponds to potential transmission events between patients. The exact time of each inpatient visit is not included in the HCUP SID due to privacy concerns. The information available for each visit includes the admission month, discharge month and length of stay. From these data, we computed the probability of each pair of inpatient visits being coincident in time and location. Let *a*_*i*_ and *d*_*i*_ denote the admission and discharge month, respectively, for the *i*^*th*^ inpatient visit. For a pair of visits *i* and *j* which occurred at the same hospital facility, we considered the following eight scenarios:

1. *a*_*i*_ = *d*_*i*_ = *a*_*j*_ = *d*_*j*_, i.e. the *i*^*th*^ and *j*^*th*^ visits took place entirely within the same month.
2. *a*_*i*_  + 1 =  *d*_*i*_  =  *a*_*j*_  =  *d*_*j*_, i.e. *i*'s visit spanned two distinct months, while *j*'s visit spanned only the month in which *i* was discharged.
3. *a*_*i*_ = *d*_*i*_ − 1 = *a*_*j*_ = *d*_*j*_, i.e. *i*'s visit spanned two distinct months, while *j*'s visit spanned only the month in which *i* was admitted.
4. *a*_*i*_ = *d*_*i*_ = *a*_*j*_ + 1 = *d*_*j*_, i.e. *j*'s visit spanned two distinct months, while *i*'s visit spanned only the month in which *j* was discharged.
5. *a*_*i*_ = *d*_*i*_ = *a*_*j*_ = *d*_*j*_ − 1, i.e. *j*'s visit spanned two distinct months, while *i*'s visit spanned only the month in which *j* was admitted.
6. *a*_*i*_ + 1 = *d*_*i*_ = *a*_*j*_ + 1 = *d*_*j*_, i.e. both *i*'s and *j*'s visits spanned two distinct months, and both visits were admitted in the same month and discharged in the same month.
7. *a*_*i*_ + 1 = *d*_*i*_ = *a*_*j*_ = *d*_*j*_ − 1, i.e. both *i*'s and *j*'s visits spanned two distinct months, and *j* was admitted in the same month that *i* was discharged.
8. *a*_*i*_ = *d*_*i*_ − 1 = *a*_*j*_ + 1 = *d*_*j*_, i.e. both *i*'s and *j*'s visits spanned two distinct months, and *i* was admitted in the same month that *j* was discharged.

Graphically, these scenarios can be viewed as shown in Figure A2.

Let *N*_*k*_ denote the number of days in the *k*^*th*^ earliest month of the set {*a*_*i*_, *d*_*i*_, *a*_*j*_, *d*_*j*_} according to the calendar year; e.g. if *i* was admitted in March and discharged in April, *j* was admitted in February and discharged in March, then *N*_1_ = 28, *N*_2_ = 31 and *N*_3_ = 30. Let ℓ_*i*_ denote the length of stay of the *i*^*th*^ inpatient visit. Then assuming that any day in a month is just as likely to be the admission date as any other day in the same month, the probability that two visits *i* and *j* were coincident can be computed in the following way:

- *Scenario 1:*
- *Scenario 2:*
- *Scenario 3:*
- *Scenario 4:* Similar to *Scenario 2*.
- *Scenario 5:* Similar to *Scenario 3.*
- *Scenario 6:* Always equal to 1.
- *Scenario 7:* If *N*_1_ + *N*_2_ − ℓ_*j*_ + 2 ≤ *N*_1_ +  ℓ_*i*_ − 1, then , else 0.
- *Scenario 8:* Similar to *Scenario 7.*

Using these rules, we constructed a network for all HCFO-CDIs as determined by the decision tree in Figure A1. Controls matched to our cases which were taken from the pool of potential controls identified by the decision tree were also included in the graph. However, visits corresponding to controls were assigned zero out-degree, i.e. no edges emanated from any control as they had no CDI diagnosis. All visits belonging to the other five classifications shown in Figure A1 were included in the network. Community-acquired infections were assigned zero in-degree, as they acquired CDI outside of the hospital. Readmissions had an in-degree of 1, with this single unit of in-degree attributed to the prior CDI visit corresponding to their readmission visit. For community-onset healthcare-associated infections, we computed their in-degree using all visits preceding the index visit which took place entirely (i.e. both admission and discharge) within the prior 4 weeks; for simplicity, we assumed independence between the prior visits in this computation. These previous visits were not used when computing their out-degree.

The computation of in-degree and out-degree was most involved for transfer patients. These patients' consecutive visits were viewed as a single visit involving two facilities; however, we separately computed the in-degree and out-degree for the two visits, respecting the facilities involved for each visit. Each transfer patient was assigned to one of six possible cases based on CDI diagnoses and indication of CDI being present on admission (POA) (either through a specific present on admission tag associated with the CDI diagnosis or by the CDI being a primary diagnosis). These cases, detailed in Table A1, dictated how we computed the in-degrees and out-degrees for the two visits comprising the transfer. For example, the first row of the table represents the case where a transfer patient had no CDI diagnosis in his/her first visit and then had CDI diagnosed as present on admission in his/her second visit. Such a patient only had the potential for non-zero in-degree in his/her first visit, and only had the potential for non-zero out-degree in his/her second visit.

**Table A1.** The six possible cases for transfer patients along with the visits that their in-degrees and out-degrees could be non-zero for

Visit 1	Visit 2	In-degree	Out-degree
No CDI	CDI, POA	Visit 1	Visit 2
No CDI	CDI, not POA	Visits 1 and 2	Visit 2
CDI, POA	CDI, POA	Neither visit	Visits 1 and 2
CDI, POA	CDI, not POA	Neither visit	Visits 1 and 2
CDI, Not POA	CDI, POA	Visit 1	Visits 1 and 2
CDI, Not POA	CDI, not POA	Visit 1	Visits 1 and 2
